# Accelerating antibiotic discovery through artificial intelligence

**DOI:** 10.1038/s42003-021-02586-0

**Published:** 2021-09-09

**Authors:** Marcelo C. R. Melo, Jacqueline R. M. A. Maasch, Cesar de la Fuente-Nunez

**Affiliations:** 1 Machine Biology Group, Departments of Psychiatry and Microbiology, Institute for Biomedical Informatics, Institute for Translational Medicine and Therapeutics, Perelman School of Medicine, University of Pennsylvania, Philadelphia, PA USA; 2 Departments of Bioengineering and Chemical and Biomolecular Engineering, School of Engineering and Applied Science, University of Pennsylvania, Philadelphia, PA USA; 3 Penn Institute for Computational Science, University of Pennsylvania, Philadelphia, PA USA; 4 Department of Computer and Information Science, School of Engineering and Applied Science, University of Pennsylvania, Philadelphia, PA USA

**Keywords:** Drug discovery, Machine learning

## Abstract

By targeting invasive organisms, antibiotics insert themselves into the ancient struggle of the host-pathogen evolutionary arms race. As pathogens evolve tactics for evading antibiotics, therapies decline in efficacy and must be replaced, distinguishing antibiotics from most other forms of drug development. Together with a slow and expensive antibiotic development pipeline, the proliferation of drug-resistant pathogens drives urgent interest in computational methods that promise to expedite candidate discovery. Strides in artificial intelligence (AI) have encouraged its application to multiple dimensions of computer-aided drug design, with increasing application to antibiotic discovery. This review describes AI-facilitated advances in the discovery of both small molecule antibiotics and antimicrobial peptides. Beyond the essential prediction of antimicrobial activity, emphasis is also given to antimicrobial compound representation, determination of drug-likeness traits, antimicrobial resistance, and *de novo* molecular design. Given the urgency of the antimicrobial resistance crisis, we analyze uptake of open science best practices in AI-driven antibiotic discovery and argue for openness and reproducibility as a means of accelerating preclinical research. Finally, trends in the literature and areas for future inquiry are discussed, as artificially intelligent enhancements to drug discovery at large offer many opportunities for future applications in antibiotic development.

## Abstract (web summary)

Melo, Maasch and de la Fuente-Nunez review the current practices in use of artificial intelligence in the discovery of antibiotics and antimicrobials. They also provide details about the best-practices that should be engaged with during computational drug discovery, including open science and reproducibility.

## Introduction

Antimicrobial resistance (AMR) in clinically significant bacteria is undermining the efficacy of existing antibiotics, incurring concerning levels of global morbidity and mortality^1^. The Centers for Disease Control and Prevention estimates that 2.8 million infections are caused by antibiotic-resistant bacteria in the United States annually, leading to 35,000 deaths from such untreatable infections^2^. Current evidence also suggests that the solution may be part of the problem itself: antibiotics have been shown to cause significant damage to the gut microbiome, reducing species diversity and encouraging the evolution and dissemination of AMR genes^3^. Antibiotics under clinical trial are generally analogs to existing drugs for which AMR mechanisms have already emerged^1^, further underscoring the need for novel approaches in antibiotic discovery.

Compounding this issue, antibiotic development is a slow, expensive, and failure-prone process that can span over 10 years and cost hundreds of millions of dollars^4^. Between 2014 and 2019, only 14 new antibiotics were developed and approved^5^. In a survey of nearly 186,000 clinical trials for over 21,000 compounds, the probability of success for new drugs that treat infectious diseases was 25.2%^6^. For orphan drugs, i.e., those that treat rare infectious diseases, this probability dropped to only 19.1%^6^. This risk of failure drives corporations to pursue research and development with a higher guarantee of return on investment, opening the way for academia to initiate early stages of antibiotic design and optimization^7,8^.

Accelerated antibiotic discovery will require computer-aided prospection for novel drugs with new mechanisms of action (MOAs)^9^. It is speculated that 10^30^–10^60^ drug-like chemicals exist^10^, while 20^*n*^ variants exist per *n*-length canonical amino acid sequence. Although this immense combinatorial space presents a broad opportunity for computational antibiotic design, an exhaustive search cannot be achieved on a reasonable timescale. These challenges strongly incentivize the development of efficient heuristics and artificially intelligent algorithms for high-throughput antibiotic discovery. A prominent subdomain of computer science, artificial intelligence (AI) concerns the study and development of machines that are capable of learning, problem-solving, or mimicking other displays of reasoning akin to natural intelligence. For the purposes of this review, AI will generally pertain to machine learning (ML), the training of mathematical models to output predictions when presented with previously unseen data. The application of ML to drug discovery, and antibiotic discovery specifically, has been greatly facilitated by the public availability of empirical datasets (Table 1), advances in computer engineering, and the proliferation of free and open-source ML libraries.

**Table 1:** Databases for computational antibiotic discovery.

Database	Site
General drug discovery and biomolecular informatics	
Binding MOAD^160^	https://bindingmoad.org
BindingDB^161^	https://www.bindingdb.org/
BRENDA^162^	https://www.brenda-enzymes.org
ChEMBL^163^	https://www.ebi.ac.uk/chembl/
Drug Design Data Resource	https://drugdesigndata.org
Drug Repurposing Hub^140^	https://clue.io/repurposing
DrugBank^164^	https://go.drugbank.com
MoleculeNet^165^	http://moleculenet.ai
Protein Data Bank^166^	https://www.wwpdb.org
PubChem^167^	https://pubchem.ncbi.nlm.nih.gov
Search Tool for Interacting Chemicals^168^	http://stitch.embl.de
Side Effect Resource^169^	http://sideeffects.embl.de
SuperTarget^170^	http://insilico.charite.de/supertarget/
Therapeutics Data Commons	https://zitniklab.hms.harvard.edu/TDC
Therapeutic Target DB^171^	http://db.idrblab.net/ttd/
UniProt^172^	https://www.uniprot.org
ZINC^173^	https://zinc15.docking.org
Exclusively infectious disease	
ADAM^174^	http://bioinformatics.cs.ntou.edu.tw/adam/
ADAPTABLE^175^	http://gec.u-picardie.fr/adaptable
Collection of Antimicrobial Peptides^176^	http://www.camp.bicnirrh.res.in
Data Repository of Antimicrobial Peptides^177^	http://dramp.cpu-bioinfor.org
DB of Antimicrobial Activity and Structure of Peptides^178^	https://dbaasp.org
dbAMP^179^	http://140.138.77.240/~dbamp
MEGARes: Antimicrobial DB for High-Throughput Sequencing^180^	https://megares.meglab.org
National DB of Antibiotic-Resistant Organisms	https://www.ncbi.nlm.nih.gov/
Pathosystems Resource Integration Center^181^	https://www.patricbrc.org
Tropical Disease Research Targets^182^	https://tdrtargets.org

Public databases (DB) of general use in computational drug discovery and biomolecular informatics, as well as those specific to antimicrobial discovery and resistance.

The integration of computational tools to expedite drug development has led to key advances for the rational design of bioactive compounds efficacious in animal models, thus demonstrating that computers can yield preclinical antibiotic candidates^9,11^. Leveraging advances in protein structure prediction and modeling, small-molecule antibiotic targets can be reliably described in atomic detail. Protein structures are then probed for binding sites, allowing large libraries of compounds to be used for automated large-scale docking and binding affinity studies in a process known as virtual screening (VS). This practice is now integral to many drug development pipelines, receiving ample attention from the ML community^12^. The most challenging step in VS is evaluating binding site affinity, driving the development of ML tools that significantly outperform traditional binding affinity prediction methods^13–15^. In recent years, deep learning (DL) has been used to successfully bypass docking and affinity estimation entirely, resulting in the identification of a small-molecule antibiotic active against multiple bacterial pathogens^16^.

In this review, we will focus on the application of AI to the development of two major classes of bioactive compounds: small-molecule antibiotics and antimicrobial peptides (AMPs). The former, studied since the beginning of the twentieth century with the discovery of penicillin and in use for over 70 years, represents the majority of antibiotics in use today. The latter, a class of small proteins usually composed of 5 to 50 amino acids, is receiving increasing attention in research and clinical trials^17^ due in part to a relatively low propensity to induce AMR^18^. Research topics will be introduced by following the logical flow of an ML pipeline, starting with compound representation and progressing through trait prediction and novel compound design. ML innovation in general drug development will be reviewed where it has cross-over utility for antibiotic-specific applications. Trends in the literature and directions for future research will be discussed, including prospects for increasing data availability, computational–experimental collaboration, and innovation in interpretable ML (IML). Additionally, we provide an original analysis of open science practices among cited research and discuss the potential for best practices in open and reproducible ML to expedite antibiotic discovery.

## Methods for optimizing compound representation

**Fig. 1:**
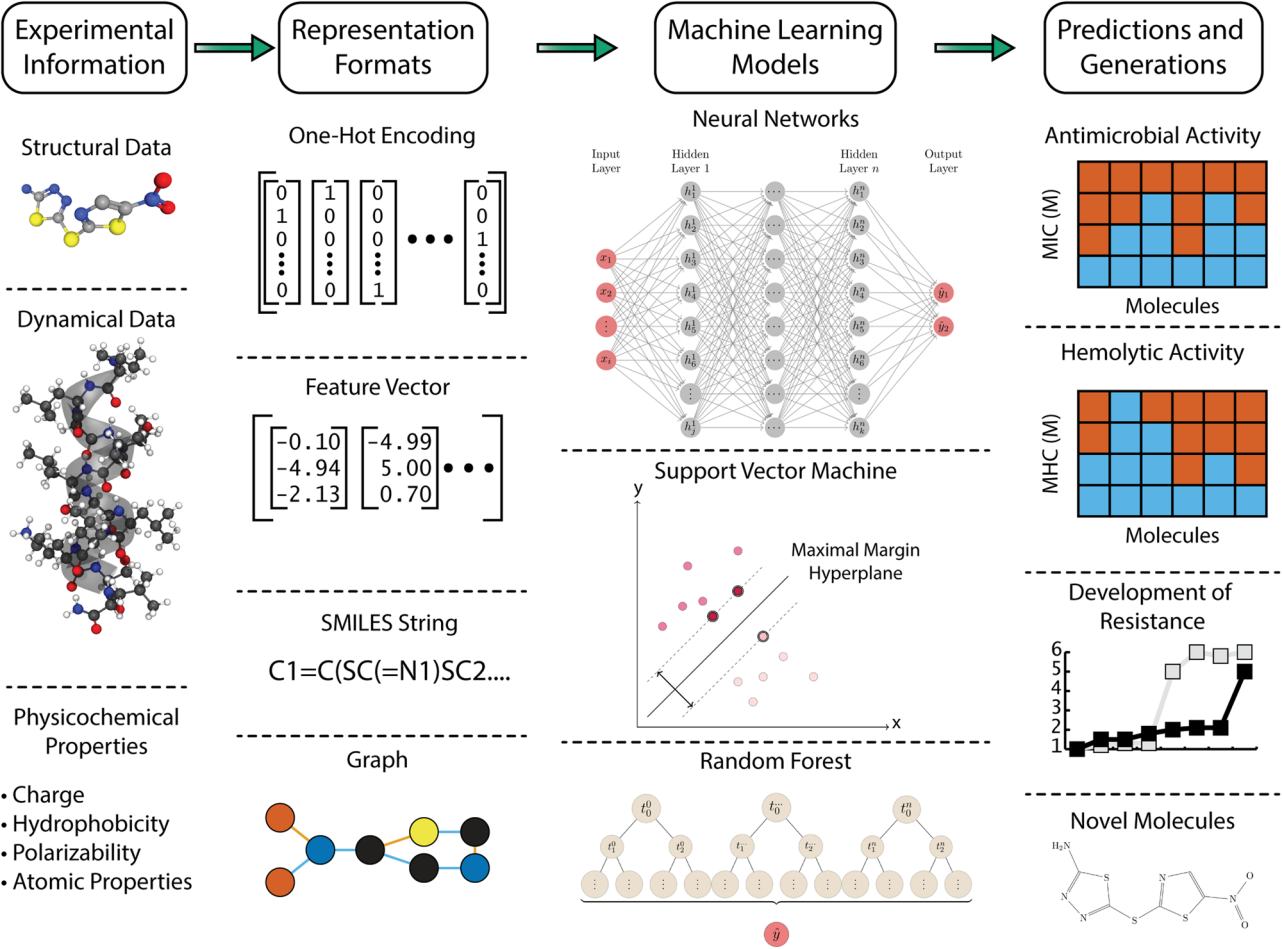
Computational antibiotic discovery pipeline. The figure provides an overview of data and methods used in antibiotic discovery and development using AI. From left to right, key elements in the drug development process are exemplified. The first part of any AI-driven project is gathering the experimental information that will enable model creation. The data are then transformed into AI-ready representations. Subsequently, models are trained using algorithms that can range from traditional decision trees to novel neural networks. Finally, trained models can be used to predict diverse qualities, e.g., the effectiveness of an antibiotic, potential for toxic activity, development of resistance, or the structure of novel compounds that exhibit desirable traits.

The search for optimal measurements of quantitative structure–activity relationships (QSAR) drove over 50 years of research and innovation^19^. Aiming to computationally predict the activity of newly designed molecules, saving time and money by avoiding synthesis and experimentation on inactive compounds, researchers relied on computational representations of drug candidates to predict their properties. As it became apparent, the problem of representing biological or chemical data for use in computational models is in itself an important field of research. Likewise, it is an essential component of the computational drug discovery pipeline (Fig. 1). The variety of information sources and experimental procedures to describe molecules can rapidly lead to overwhelming amounts of information, which may cause more harm than good. For example, in order to describe simple amino acid residues, over 400 different measurements have been performed and combined in online databases^20^. For small-molecule drugs, approaches range from calculating and condensing quantum mechanically derived descriptors^21^ to calculating topological properties^22,23^. The sheer amount of data and the redundant information contained in multiple measurements makes using all descriptors impractical or counterproductive. This led to a series of studies that combined experimental data into reduced descriptors that maximized information content in as few dimensions as possible^24^.

From traditional dimensionality reduction techniques like principal component analysis (PCA) and singular value decomposition to feature selection approaches involving *χ*^2^ statistical tests or mutual information estimation, the search for reduced and information-rich representations has now fully integrated ML tools and principles. The efforts described below highlight how the theoretical and methodological advances made in diverse ML applications and interest areas can be adapted to aid ML-driven antibiotic development.

A prominent example is the use of graph convolutional networks to leverage the geometry and connectivity of molecules to naturally translate them into graphs, using neural networks to learn from the chemical structure itself^25^. A similar approach was taken to study and predict protein structures^26^. In an extensive benchmark study of available methods and datasets^27^, it was found that neural networks can enhance not only the process of describing a drug based on a set of molecular descriptors but also the determination of such molecular descriptors themselves. This work was extended to create a series of antimicrobial compounds that were correctly predicted as active despite being structurally distant from known antibiotics ^16^.

While common in the fields of signal processing and natural language processing (NLP), recurrent neural networks (RNNs) have now been adapted to process simplified molecular-input line-entry system (SMILES) representations, which encode structures of chemical species using simple text strings. In one case, researchers used long short-term memory (LSTM) generative neural networks to learn from SMILES representations of known drugs and then used the trained neural network to generate new compounds^28^. Alternatively, RNNs have been combined with reinforcement learning to autonomously create an embedded representation for drugs based on their SMILES representations^29^.

RNNs have emerged as a natural embedding approach for AMP sequences, given their ability to parse sequence-based inputs. Based on a one-hot encoding of amino acid residues (i.e., a 20-mer vector with 19 “zeros” and a single “one” at unique positions to indicate different residues), both an LSTM-based autoencoder^30^ and multiplicative-LSTM neural network^31^ have been trained to create embedded representations for peptide sequences. The latter led to an embedded representation that could be used to derive a protein’s secondary structure, thermal stability, deep mutational scanning classification, and even the functional impact of mutations^31^.

## Antimicrobial activity prediction

Predicting antimicrobial activity is at the core of ML integration into antibiotic development, driving over 10 years of research to provide new solutions for the QSAR problem^7^ and attracting a variety of approaches^32^ (Table 2). For instance, to improve upon previous attempts to design new drugs based on the analysis of chemical fragments and their properties, researchers used multinomial logistic regression to classify fragments that comprise molecules in a training set. This process created a “vocabulary” of fragments that could then be combined to propose new antibiotics active against the Gram-negative bacterium *Pseudomonas aeruginosa*^33^.

**Table 2:** Machine learning models for antibiotic discovery.

	Public release			
Algorithm	Code	Data	Software	Software type
Antimicrobial activity prediction				
Artificial neural network^40^		Yes		
Support vector machine^38^		Yes		
Multinomial logistic regression^33^		Yes		
LSTM RNN^44^	Yes	Yes	Yes	Command-line tool
XGBoost^42^	Yes	Yes	Yes	Command-line tool
Directed-message passing neural network^16^	Yes	Yes	Yes	Web server, Docker container
DBSCAN^47^		Yes	Yes	Web server
DBSCAN^48^			Yes	Web server
Convolutional neural network^41^		Yes	Yes	Web server
Generalized linear model^49^				
Random forest^50^				
Hemolytic activity prediction				
Classification and regression trees^55^		Yes		
Artificial neural network^54^		Yes	Yes	Web server
Gradient boosting classifiers^56^	Yes	Yes		
Support vector machine^183^		Yes	Yes	Web server, mobile app, standalone
De novo antibiotic design				
Variational autoencoder^45^		Yes		
LSTM RNN^30^	Yes	Yes	Yes	Command-line tool
LSTM RNN^120^				
Generative adversarial network^119^	Yes	Yes	Yes	Command-line tool

Machine learning models cited in this review pertain specifically to antimicrobial compound discovery, i.e., those that predict antimicrobial activity, those trained on antimicrobial compound data to predict drug-likeness, and those that generate potential antimicrobials. Public release of model source code, training and/or testing data, and/or associated software tools are noted. Criteria for data release were lenient, with “yes” indicating partial or full release of training or testing data.

In a recent effort to repurpose previously developed drugs as antibiotics^16^, a combination of neural network models was used to create a new representation for chemical compounds, and then assess their antimicrobial potential. Interestingly, this effort also made use of *ensemble learning*^34^, a technique that combines multiple copies of a model (with different weights or architectures) and takes a weighted vote of each model into consideration to achieve the final prediction^35^. The underlying assumption behind ensemble learning is that errors made by one model will be compensated for by others, and this assumption has been confirmed in applications ranging from proinflammatory peptide identification^36^ to prediction of drug side effects^37^.

Classical ML techniques such as support vector machines (SVMs) have been applied to describe AMPs and quantify their MOAs^38,39^. Alternatively, deep neural networks have been used to predict antimicrobial properties from simplified residue representations of arbitrary amino acid sequences. In 2009, researchers combined 44 peptide descriptors traditionally used for QSAR studies and used them as inputs for an artificial neural network that predicted peptide activity against *P. aeruginosa*^40^. More recently, a 2020 study created a deep convolutional neural network model based on a simplified amino acid vocabulary that translated the natural 20 amino acids into pseudo residue types^41^. This model predicts antimicrobial activity in small peptides and is available in a web server. Extreme gradient boosting has been used for genome-based prediction of minimum inhibitory concentrations for 20 antibiotics against *Klebsiella pneumoniae*^42^ and 15 antibiotics against nontyphoidal *Salmonella* strains^43^. Using RNNs^44^, a combination of input representation and regression models were created to select peptide sequences with antimicrobial activity. Finally, through a variational autoencoder approach, peptide sequences were embedded in a latent space that was subsequently searched for new AMP sequences^45^.

The variety of techniques utilized thus far correlates with an increasing focus on AMPs, which have been regarded as a major source of new antibiotics to tackle the development of resistance in microbes^9^. The ability of AMPs to limit AMR development has been related to their varied MOAs^46^, which has led researchers to focus on classifying peptides and discovering new MoAs. Specifically, DBSCAN was used for cluster-based prediction of AMP activity against Gram-negative bacteria^47^, with promising candidates being synthesized and tested in vitro^48^.

The direct combination of experimental and ML techniques in a closed-loop approach has also benefited the development of new AMPs. Starting from a template with known antimicrobial activity and a series of homologous sequences, it was possible to train a generalized linear model to create new AMPs with 160-fold increased antimicrobial activity against *Escherichia coli*^49^. Since patterns found by generalized linear models can be directly interpreted by analyzing the model weights, one can directly translate the model into actionable information for AMP design.

While most ML-based antibiotic development approaches focus on creating new representations for drug candidates and new models to predict their activity based on molecular descriptors, the phenotypic drug discovery approach focuses not on describing the molecule itself but on its effects on target organisms. For example, a recent study used a random forest model to predict antimicrobial activity based on featurization of cell imaging, avoiding detailed description of the molecules themselves^50^. This approach can expand the search space for new drugs by avoiding direct comparisons between molecular descriptors and focusing instead on their effects on pathogens.

## Drug-likeness prediction

ML can yield a fuller aggregate picture of antibiotic therapeutic potential than simply predicting antimicrobial activity. Attempts to quantitatively distinguish the subsets of chemical space that have therapeutic potential from those which do not have yielded various schemas, including the introduction of the Rule of 5 in 1997^51^ and subsequent concepts of drug-likeness and lead-likeness. Prediction of drug-likeness has been refined and increasingly automated over recent decades, with traits of interest including absorption, distribution, metabolism, excretion, and toxicity (ADMET)^10,52^. ML-based prediction of binding affinity can also accelerate high-throughput screening and structure-based drug lead optimization by pinpointing candidates with more favorable drug–target interactions, as discussed in recent reviews^15,53^.

Like many ML problems, drug-likeness prediction can be attempted using a wide array of algorithms. While experimental observations often require a specific methodology or well-established gold standard, diverse ML algorithms can often provide comparable performance for a given classification or regression problem. There is often no way to know a priori which algorithm will perform best, although theoretical knowledge can guide decision-making. Therefore, it is important to follow a rigorous model selection process that compares multiple algorithms (e.g., a Gaussian process, random forest, SVM, and neural network) across several performance metrics that are salient to the particular use case. In this section, we note the use of diverse algorithms that have been applied to multiple drug-likeness prediction problems.

Dangerous pharmacokinetic properties and toxicity are leading causes of clinical trial failure^52^, incentivizing pre-trial in silico exploration. Host cell toxicity is a critical ADMET endpoint and a significant risk in antibiotic development, motivating the design of predictive tools for mammalian red blood cell toxicity, kidney cell toxicity, and other forms of eukaryotic cell damage. Hemolytic activity, or the ability to burst red blood cells, has been a major focus of therapeutic development given that numerous drugs enter the bloodstream. Prediction of hemolytic activity in AMPs and antimicrobial peptidomimetics has been explored using neural networks^54^, classification trees^55^, and gradient boosting classifiers^56^. Consensus model-based software for hemolytic activity prediction has also been released for general applications in drug development, with an emphasis on small molecules^57^ and saponins^58^. A feedforward fully connected neural network has demonstrated comparable performance to prior random forest models for the prediction of drug candidate cytotoxicity^59^. Deep Taylor Decomposition was used to identify the most significant features in DL-based cytotoxicity classification, with an emphasis on visualization to facilitate interpretability^59^. Additional antibiotic side effects can also be foreshadowed using ML, as has been done for the seizure-inducing potential of enoxacin, a broad-spectrum fluoroquinolone antibacterial^60^.

The development of AMP-based antibiotics must also consider peptide solubility and stability, which are necessary for manufacture and efficacy. Pharmaceutically viable AMPs will be soluble, a trait that can be predicted from amino acid sequence^61^. Protein solubility prediction has used neural network^61,62^, gradient boosting machine^63^, logistic regression classifier^64^, SVM^65^, and random forest models^66^. Degradation via the action of proteolytic enzymes is a significant concern when evaluating the stability of peptide-based antibiotics^67,68^. The in silico identification of putative proteolytic cleavage sites can inform AMP lead selection and guide sequence optimization for increased stability. Cleavage site prediction has been explored through the lens of drug development^69^ and other protein informatics applications using classification and regression mode SVM^70–74^, convolutional neural network^75^, conditional random field classifier^76^, and logistic regression models^77^. Similarly, the stability of drug-like chemicals has been modeled using an attention-based graph convolution neural network^78^ and Naive Bayes classifier^79^.

As outliers to original drug-likeness definitions expand the boundaries of these criteria, new qualitative endpoints and quantitative thresholds have come under consideration^80^. Collateral damage to the gut microbiome has been proposed as one additional ADMET endpoint, and consensus model-based software has been released for ML prediction of microbiome damage^58^. Indeed, disruption to the microbiome is a significant side effect of antibiotics and has been implicated in AMR evolution^3^. For this particular endpoint, species-specific antimicrobial activity prediction may be the answer: ML can aid in the selection of candidates with high specificity for target pathogens and low activity against known commensals.

## AMR prediction

Unlike most therapeutics, antibiotics are designed to kill a living target with the capacity for resistance evolution. The near-inevitability of AMR evolution thus adds an additional urgent consideration that is absent from most other drug development niches. Incentives to develop less resistance-prone countermeasures are drawing research to historically underexplored sources of inspiration for novel antibiotic design^9^. Likewise, the need to track AMR emergence, mechanisms, and dynamics are raising new applied ML questions unique to computational antibiotic discovery, bacterial genomics, and infectious disease epidemiology. While ML-based AMR prediction may be clinically useful for informing AMR diagnosis and antibiotic prescription^81,82^, it may also be experimentally useful in the drug development process. We anticipate that ML approaches to AMR genomics in epidemiology and medicine will increasingly be adapted specifically for drug development purposes, e.g. ML-informed resistance evolution experiments for new lead compounds.

Protein space is one such underexplored area that is expected to yield future antibiotics with minimal AMR risk. Antimicrobial host defense peptides, including encrypted AMPs released from precursor proteins through proteolytic cleavage, have notably emerged as reservoirs for low AMR-risk antibiotic templates due in part to a tendency to act on multiple cellular targets^18,46,83,84^. Small-molecule AMR has also been observed to coincide frequently with a collateral sensitivity to AMPs, yet rarely with AMP cross-resistance^85^. Together with the fact that protein target modifications are a common AMR mechanism, this suggests the large potential for cross-over between ML and traditional protein informatics in AMR research. However, the majority of existing ML models forgo this route in favor of pathogen genetic and genomic inputs. Although model design strategies are expected to diversify, the current state of ML for AMR learns from the bacterial genome rather than drug or molecular target features.

Pathogen genomic data have been used to build ML models of antibiotic susceptibility and resistance phenotypes in clinically relevant bacteria, including *K. pneumoniae*^42^, *E. coli*^86–88^, *P. aeruginosa*^86,89^, *Mycobacterium tuberculosis*^90,91^, and *Staphylococcus aureus*^86^. While ML models of AMR may be trained on drug- and bacteria-specific data^92–94^, a more agnostic approach has been explored using a neural network to facilitate environmental metagenomic analysis^95^. However, predictive performance has been observed to vary significantly by antibiotic, target species, genomic data sampling method, and resistance mechanism complexity^82,96^, suggesting that AMR prediction may at times require relatively context-specific modeling. A free web server and standalone software have been released for SVM-based prediction of efflux-mediated AMR^97^. ML-assisted metagenomic analysis has implicated AMR genes associated with antibiotic-induced microbiome perturbations^98^. A novel combination of protein homology modeling and LASSO penalized logistic regression has been used to investigate the horizontal transfer of antibiotic resistance determinants from gut commensals to bacterial pathogens^99^.

While “black-box” approaches may limit the utility of ML for AMR-risk reduction^81^, IML can enable models to suggest causal factors in AMR at the organismal and population scale. Coupling ML with gene–protein structure mapping to investigate drivers of *M. tuberculosis* AMR evolution, interactions between genes conferring AMR were hypothesized to manifest as correlations in their weights and signs across the hyperplanes of an SVM ensemble^100^. An ML-integrated genome-scale model using data from microbial genome-wide association studies has enabled allele-parameterized flux balance analysis to reveal metabolomic insights into *M. tuberculosis* AMR^101^. Open-source software using protein orthology-based gene variant mapping has also been developed for interpretable AMR prediction^96^. Computationally characterizing the molecular signatures and population dynamics of AMR might help indicate which MOAs are overused and which present promising new avenues, even on a regional scale. Using training data from multiple countries, geographic analysis of predicted AMR genes revealed population dynamics that could be supported by national rates of multidrug-resistant tuberculosis and antibiotic prescription trends^100^.

## Generative DL for antibiotic discovery

Generative DL can lend itself to computational antibiotic discovery in multiple ways. Here, we will focus on de novo molecular design, which often employs generative adversarial networks (GANs), variational autoencoders (VAEs), or related architectures. Comprised of dueling generative and discriminative models, GANs infer the probability distribution from which training data derive in order to construct novel samples from this distribution. Engaging in a two-player minimax game, both models are trained to optimize the error rate of the discriminator: while the generator is trained to maximize the likelihood that the discriminator fails to distinguish empirical data from synthetic data, the discriminator is trained to minimize this likelihood^102^. Like classical autoencoders, VAEs are trained to encode inputs to a compressed representation and then to decode an approximate reconstruction, learning the latent variables describing the training data in the process. However, VAEs are directed probabilistic models, learning continuous latent variables through a variational Bayesian approach to generative DL^103^. This section will note the use of several variations on these common generative architectures as applied to drug discovery.

Generative DL has found diverse chemical and protein engineering applications^104^, including inverse design of inorganic matter^105^ and graph-based neural network models for the NP-hard^106^ inverse protein folding problem^26,107^. Increasingly, generative DL is applied explicitly to drug discovery, whereby synthetic molecular designs are proposed from drug-like chemical spaces. De novo drug candidate design has been attempted with deep reinforcement learning coupling generative and predictive neural networks^29^, deep generative adversarial autoencoder architecture^108^, differentiable neural computer architecture with reinforcement learning and adversarial training^109,110^, deep neural networks coupled with Monte Carlo tree search^111^, and an autoencoder–GAN combination for both random and target-biased molecular design^112^. Given their suitability for sequential data, generative RNNs taking SMILES inputs have drawn attention in drug design^113,114^ and have demonstrated relatively broad, uniform, and complete coverage of chemical space^115,116^. Experimentally validated membranolytic anticancer peptides have been generated by both an LSTM RNN with transfer learning^117^ and a counterpropagation artificial neural network optimized by a genetic algorithm^118^.

A burgeoning interest in generative DL within chemical engineering, protein engineering, and drug development at large suggests that similar techniques may be increasingly applied to AMP and small-molecule antibiotic design. To date, a GAN has been used to generate an AMP with a significantly lower minimum inhibitory concentration against *E*. *coli* than ampicillin^119^. Additional preliminary success in AMP discovery is described in a proof-of-concept study coupling a VAE with experimental validation^45^. A generative LSTM RNN with transfer learning has demonstrated success in reconstructing molecules known to target *S. aureus* after pretraining on a large generalized dataset and fine-tuning on a smaller set of target-specific bioactive molecules^120^. An RNN with unidirectional LSTM cells for de novo AMP design observed 82% of generated peptides to be putative AMPs, while only 65% of random permutations from the amino acid distribution of the training data were predicted to be antimicrobial^30^.

## Openness and reproducibility

In this section, we present an argument for increasing openness and reproducibility in ML-based antibiotic discovery. This argument hinges on a two-pronged crisis: (1) the global public health crisis of AMR, slow antibiotic development rates, and emerging infectious diseases and (2) the reproducibility crisis currently plaguing AI. We conclude with an original analysis of open science practices among the publications cited in this review.

Accelerating antibiotic discovery through open information and technology exchange carries both practical and ethical weight. As evidenced by poignant examples from the COVID-19 pandemic, factors such as AMR^121^, sudden pathogen emergence, unexpected large-scale losses in quality of life and economic security^122^, and structural inequities that render some populations disproportionately vulnerable^123^ raise unique questions of urgency and justice in infectious disease control. These questions heighten the need for swift research and development, evoking calls for increased openness under global public health crises^124^. We argue that similar calls should extend to the global crisis of AMR evolution, and thus to computational antibiotic discovery.

The international movement toward open-access publishing represented by groups like cOAlition S^125^ signal a growing concern for transparency, reproducibility, and equitable access to information within the scientific community. Effective 2021, Plan S dictates that publications resulting from public and private grants of participating bodies “must be published in Open-Access Journals, on Open-Access Platforms, or made immediately available through Open-Access Repositories without embargo” (https://www.coalition-s.org/plan_s_principles/). Nevertheless, open-access publishing addresses only one facet of computational openness and reproducibility. With stakes as high as they are in computational antibiotic discovery, we call for a more comprehensive set of open science best practices.

An open science regime that ensures computational reproducibility can accelerate ML-based antibiotic discovery through free public access to (1) source code, (2) training and testing data, and (3) published findings. Computational reproducibility facilitates the external validation of published claims while encouraging the dissemination of knowledge and methods. However, standards of openness and reproducibility in biomedical ML are still subject to debate^126^, and some argue that AI generally suffers from a reproducibility crisis, not unlike that of psychology^127^. Reproducibility challenges common to ML (e.g., verbal descriptions in lieu of source code omitting essential hyperparameter values or random state seeds) can also have detrimental interactions with challenges unique to biomedicine (e.g., patient privacy laws precluding data sharing)^128^.

Although releasing source code, training data, and testing data could mitigate reproducibility concerns while increasing the scientific value of AI research^126^, an analysis of 400 general AI conference papers revealed that only 6% released code, 54% released pseudocode, and ~30% released test data^127^. Within ML for the life sciences and medicine specifically, a recent review found that 50% of 300 publications released software, while 64% released data^129^. A review of 511 studies found that papers applying ML to healthcare data underperformed relative to NLP, computer vision, and general ML on multiple metrics of reproducibility, including code release rates^130^. A systematic review of 415 studies on ML-based image analysis for COVID-19 diagnosis found that all publications contained serious methodological flaws or failed to report key information needed for reproducibility and substantiation of claims, such that not a single model was of clinical use^131^.

Confounding factors such as lack of incentives in academia or misaligned objectives in the private sector may further hinder the adoption of open science practices. While open-access journals continue to grow, many prestigious scientific journals charge premiums over publication fees in order to make articles open access. Authors then face a tough choice between funding their research or paying premiums to make their publications free to all readers. Indeed, a recent study showed that authors of open-access publications in US research institutions tend to have more access to funding and belong to more advanced career stages^132^. Exemplifying the conflict between researchers and publishers, the recent 2-year-long negotiation between the University of California system and Elsevier resulted in the largest deal for open-access publishing for scientific articles in North America^133^. Interestingly, the fields of medical and biological research are among the most accessible, with biology having the largest fraction of immediately free-to-read articles^134^.

Beyond publishing research findings, the release of source code and training and testing data may also raise conflicts regarding intellectual property (IP) and competitiveness in the private sector. Therefore, while industry-funded research for antimicrobial discovery^135^ can still provide great advances to the field, finding a balance between open access and closed IP may prove to be a barrier in itself. Guidance may be found in the efforts of related fields to establish community-wide standards for responsible and reproducible ML publications, with the Checklist for Artificial Intelligence in Medical Imaging being a notable example^136^.

**Fig. 2:**
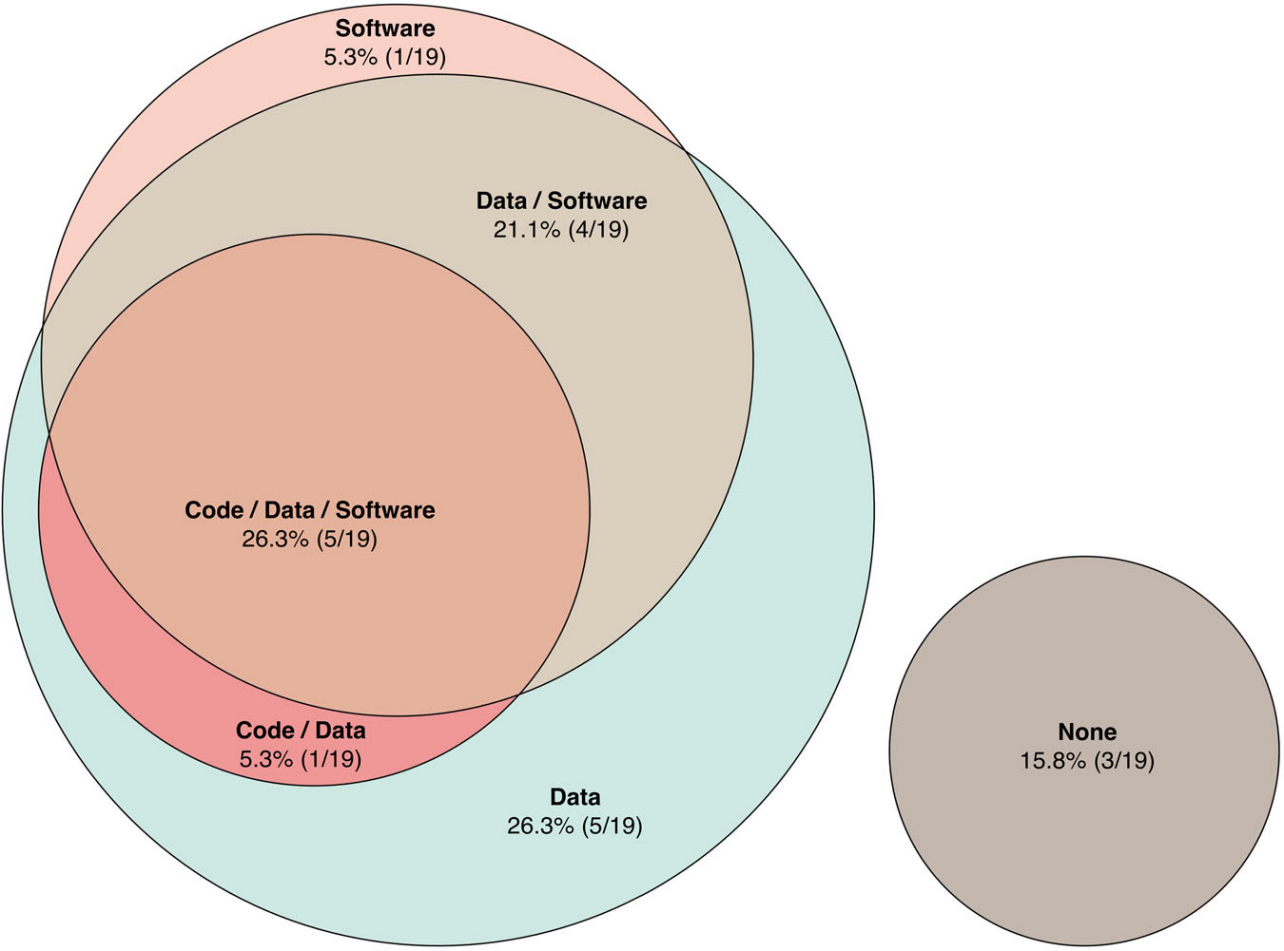
Open science practices in machine learning for antibiotic discovery. This Euler diagram visualizes public release rates for source code, training or testing data, software, and combinations thereof among publications cited in this review (Table 2). Note that data release criteria for this analysis include both partial and full public availability. This analysis was performed post hoc on studies previously cited in this review.

This conversation in AI, and in biomedical ML specifically, motivated our analysis of code, data, and software release rates among models cited in this review (Fig. 2). This analysis was performed post hoc, such that all studies previously cited in this review that presented an ML model designed for antimicrobial compound discovery were included. It, therefore, focuses on key contributions in ML-facilitated antibiotic discovery, rather than an exhaustive literature analysis. Among ML models pertaining specifically to antimicrobial compound discovery (Table 2), we found that 31.6% (6/19) released code, 52.6% (10/19) released software, and 78.9% (15/19) released some or all training or testing data. Further, 26.3% (5/19) released code, data, and software, while 15.8% (3/19) released nothing in these three categories. It should be noted that our criteria for data release were lenient, with “yes” indicating partial or full release of training or testing data. Although best practice is to release full, metadata-documented versions of both training and testing datasets in a manner that is easily accessible for the reader, this is often not the standard followed in past publications. While our sample size is small, we hope that these statistics will inspire increased best-practice public release rates in ML for antibiotic discovery.

Moving forward, inspiration can be found in projects taking a broad view of openness and reproducibility in drug discovery. The open-source Therapeutics Data Commons (https://zitniklab.hms.harvard.edu/TDC/) provides free ML datasets to lower barriers to entry and accelerate drug development pipelines. The Open-Access Antimicrobial Screening Program extends the concept of openness to experimental methods by offering free compound screening services (https://www.co-add.org). Such creative counterexamples to the closed research paradigm will ideally become the norm in antibiotic discovery.

## Trends and future directions

In this section, we examine research trends and discuss future trajectories for ML-facilitated antibiotic discovery. We anticipate that a trickle-down effect from adjacent ML research will stimulate significant AI-facilitated innovation in antibiotic discovery over the next decade. We expect this innovation process to require increased data quality and availability, exploration of new regions in chemical space, re-exploration of known regions through drug repurposing, collaboration between computational scientists and experimentalists, and enhanced explainability through IML.

**Fig. 3:**
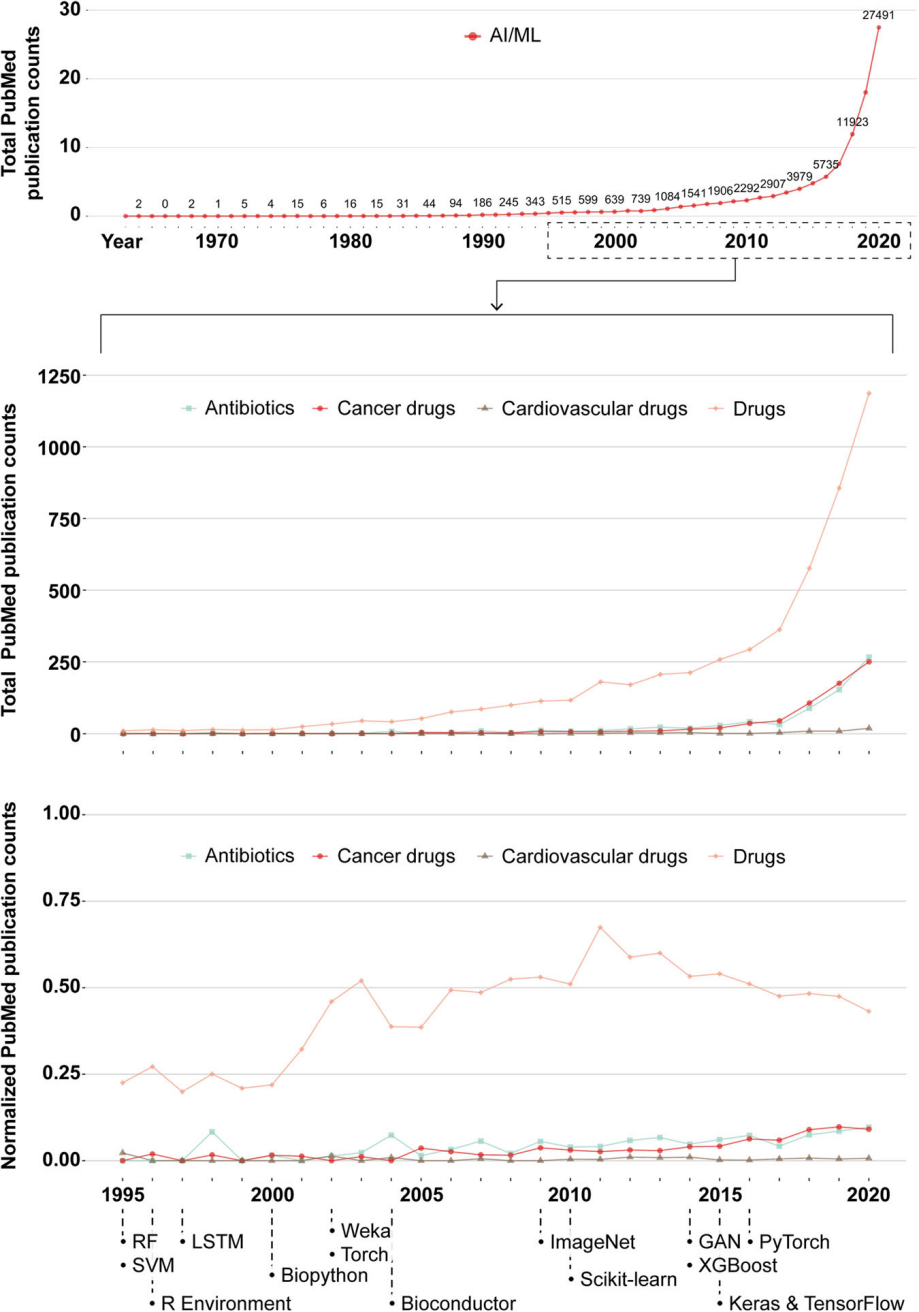
Machine learning in antibiotic discovery over time. From top to bottom: total PubMed results when querying for AI/ML keywords only, total results when querying for AI/ML and general or disease group-specific drug keywords, and the proportion of general AI/ML publications pertaining to each category of drugs (i.e., total publication counts per drug category scaled by total AI/ML publications per year). Queries sought keywords in titles and abstracts only, with the general drug query excluding keywords contained in the disease group queries to prevent double-counting. Key events in the broader ML community are noted to contextualize trend lines. The relevant literature used to set key dates are as follows: development of SVM^146^ and random forest algorithms^147^ in 1995; publication of the R language and software environment in 1996^148^; development of LSTM in 1997^149^; development of the Biopython package in 2000^150^; release of the Java interface for Weka in 2002^151^; publication of the Torch library in 2002^152^; release of Bioconductor in 2004^153^; the publication of ImageNet in 2009^154^; the initial release of Scikit-learn in 2010^155^; the initial release of XGBoost^156^ and development of GANs^102^ in 2014; development of Keras^157^ and TensorFlow^158^ in 2015; and the initial release of PyTorch in 2016^159^. Exact Boolean searches in PubMed can be found in Supplementary Table 1.

To assess the state of publishing on ML for antibiotic discovery, we measured trends among papers in PubMed, a public database maintained by the United States National Library of Medicine of the National Institutes of Health (https://pubmed.ncbi.nlm.nih.gov). To explore the extent to which research interest has changed over time, we queried PubMed by year for texts on ML and antibiotics, ML and cancer therapies, ML and cardiovascular drugs, or ML and drugs broadly defined (Fig. 3). Querying for applications of ML to broad drug development serves as a benchmark against which to compare engagement levels in antibiotic-specific applications. Disease group-specific keywords were excluded from the general drug query to prevent double-counting. As cardiovascular disease and cancer are the two leading causes of death in the United States^137^, querying for these applications provides relevant public health context for infectious disease applications. Further, a blanket query for AI and ML keywords with no additional qualifiers provides the most macroscopic view of research interest in these predictive methodologies, irrespective of application area. Exact Boolean search phrases can be found in Supplementary Table 1.

Results indicate increasing research interest in all areas over the first two decades of the twenty-first century, with the volume of ML literature focused explicitly on antibiotics and cancer drugs lagging behind broader drug development applications by nearly a decade. Surprisingly, publication counts for cardiovascular drugs and ML remain very low. As the general drug query did not double-count observations from the major disease groups explored, these results may suggest that broad applications have received greater research attention than disease group-specific applications. However, similar trends in cancer- and antibiotic-related publication rates suggest that antibiotics might not be disproportionately neglected. The prevalence of general-interest lines of inquiry might be due to the relative recency of ML for drug discovery, whereby the initial establishment phase lays the groundwork for future specialization. To that end, the significantly higher volume of general drug development applications represents a reservoir of research that is expected to have trickle-down impacts on disease group-specific research over time. Further, the proportion of AI and ML publications that feature applications in general drug discovery, antibiotic discovery, and cancer drug discovery have each increased throughout the twenty-first century. Our analysis also marks 2018 as a watershed moment for the use of ML for antibiotic discovery, coinciding with landmark papers in the field published that year together with preceding software developments.

Over the third decade of the twenty-first century, prospects for ML-facilitated antibiotic discovery will partially hinge on data improvements. As larger data sources become publicly available, new ML questions can be pursued and ongoing questions can be revisited with greater rigor. While expanding public sources of experimental data will be crucial, federated learning across institutions may facilitate empirical dataset expansion without sharing private data, as has been done in other areas of biomedical ML^138^. Increased data sharing from both successful and failed projects in the pharmaceutical industry has also been proposed as a means of accelerating research and development^139^. Existing data can also be further mined for new purposes, as exemplified by resources like the Drug Repurposing Hub^140^. While ML increasingly opens up new regions of chemical space to exploration, the repurposing of non-antibiotic pharmaceuticals could also be a promising avenue for antibiotic discovery^1^ that has already benefited from DL methods^16^.

A recent review observed greater technical correctness among biomedical ML publications featuring collaborations across computer science, biology, and medicine^129^, suggesting that computational antibiotic discovery might similarly benefit from combined expertise. Increased coupling of in silico model testing with in vitro and in vivo validation—and even additional computational methods, e.g., molecular dynamics simulation^141^—will help ensure that published models are robust and yield experimentally actionable predictions. Interdisciplinary collaboration might also facilitate increasingly insightful predictions through biologically informed IML. As a response to the prevalent “black-boxing” of ML models’ internal decision-making, IML is an expanding focus in biomedical computation^142^ that has been used to elucidate antibiotic MOAs^143^. As firmer terminological and methodological standards alleviate significant confusion surrounding its diverse implementations^144^, IML is expected to enable greater human interpretability and causal inference in antibiotic discovery than opaque algorithms generally allow. Expanding interpretability for causal biological insights will surely require both computational creativity and biomedical domain knowledge.

Additional new avenues for ML-facilitated antibiotic discovery are expected to trickle in from algorithmic theory, robotic AI, and adjacent computational domains. While this review has focused on ML rather than embodied AI, recent attempts to deploy intelligent robots in chemical experimentation^145^ may indicate the utility of ML-guided autonomous robotics in future antibiotic discovery. Creative integration of diverse lessons from NLP, computer vision, generative DL, computer-aided drug design, and other flourishing areas in ML research will play important roles in accelerating the urgent task of novel antibiotic discovery.

## Supplementary information

Supplementary Information

Description of Additional Supplementary Files

Supplementary Data 1

## Data Availability

CSV files containing the raw PubMed data outputs visualized in Fig. 3 are available in Supplementary Data 1. A README file containing resource metadata is also provided.
