# Supplementary material for: Accelerating antibiotic discovery through artificial intelligence
Source: Commun Biol. 2021 Sep 9;4:1050. doi: 10.1038/s42003-021-02586-0 (PMC8429579; doi:10.1038/s42003-021-02586-0)
Supplement: Supplementary file 1 — Supplementary Information [file 42003_2021_2586_MOESM1_ESM.pdf]

Accelerating antibiotic discovery through artificial intelligence

Supplementary Information

Marcelo C. R. Melo,[†,‡,¶,∥] Jacqueline R. M. A. Maasch,[†,‡,¶,§,∥] and Cesar de la Fuente-Nunez[*,†,‡,¶]

†*Machine Biology Group, Departments of Psychiatry and Microbiology, Institute for Biomedical Informatics, Institute for Translational Medicine and Therapeutics, Perelman School of Medicine, University of Pennsylvania, Philadelphia, Pennsylvania, United States of America.*

‡*Departments of Bioengineering and Chemical and Biomolecular Engineering, School of Engineering and Applied Science, University of Pennsylvania, Philadelphia, Pennsylvania, United States of America.*

¶*Penn Institute for Computational Science, University of Pennsylvania, Philadelphia, Pennsylvania, United States of America.*

§*Department of Computer and Information Science, University of Pennsylvania School of Engineering and Applied Science, Philadelphia, PA 19104, United States of America.*

∥*Both authors contributed equally to this manuscript.*

E-mail: cfuente@upenn.edu

Supplementary Table 1: Exact PubMed Boolean queries used to generate trendlines visualized in Figure 2.

Trendline	Boolean phrase
Drugs	(((drug[Title/Abstract]) OR (drugs[Title/Abstract])) AND ((artificial intelligence[Title/Abstract]) OR (machine learning[Title/Abstract]) OR (deep learning[Title/Abstract]) OR (support vector[Title/Abstract]) OR (random forest[Title/Abstract]) OR (neural network[Title/Abstract]))) NOT ((infection[Title/Abstract]) OR (antibiotic[Title/Abstract]) OR (antibiotics[Title/Abstract]) OR (antimicrobial[Title/Abstract]) OR (antimicrobials[Title/Abstract]) OR (antibacterial[Title/Abstract]) OR (antibacterials[Title/Abstract])) NOT ((cancer[Title/Abstract]) OR (cancer drug[Title/Abstract]) OR (cancer drugs[Title/Abstract]) OR (cancer treatment[Title/Abstract]) OR (radiation therapy[Title/Abstract]) OR (cancer chemotherapy[Title/Abstract])) NOT ((cardiovascular[Title/Abstract]) OR (stroke[Title/Abstract]) OR (heart[Title/Abstract]) OR (cardiovascular drug[Title/Abstract]) OR (cardiovascular drugs[Title/Abstract]) OR (cardiovascular agent[Title/Abstract]) OR (cardiovascular agents[Title/Abstract]) OR (anticoagulant[Title/Abstract]) OR (anticoagulants[Title/Abstract]) OR (antiplatelet agent[Title/Abstract]) OR (antiplatelet agents[Title/Abstract]) OR (thrombolytic agent[Title/Abstract]) OR (thrombolytic agents[Title/Abstract]) OR (beta blocker[Title/Abstract]) OR (beta blockers[Title/Abstract]) OR (vasodilator[Title/Abstract]) OR (vasodilators[Title/Abstract]))
Antibiotics	(((antibiotic[Title/Abstract]) OR (antibiotics[Title/Abstract]) OR (antimicrobial[Title/Abstract]) OR (antimicrobials[Title/Abstract]) OR (antibacterial[Title/Abstract]) OR (antibacterials[Title/Abstract])) AND ((artificial intelligence[Title/Abstract]) OR (machine learning[Title/Abstract]) OR (deep learning[Title/Abstract]) OR (support vector[Title/Abstract]) OR (random forest[Title/Abstract]) OR (neural network[Title/Abstract])))
Cancer	(((cancer drug[Title/Abstract]) OR (cancer drugs[Title/Abstract]) OR (cancer treatment[Title/Abstract]) OR (radiation therapy[Title/Abstract]) OR (cancer chemotherapy[Title/Abstract])) AND ((artificial intelligence[Title/Abstract]) OR (machine learning[Title/Abstract]) OR (deep learning[Title/Abstract]) OR (support vector[Title/Abstract]) OR (random forest[Title/Abstract]) OR (neural network[Title/Abstract])))
Cardiovascular	(((cardiovascular drug[Title/Abstract]) OR (cardiovascular drugs[Title/Abstract]) OR (cardiovascular agent[Title/Abstract]) OR (cardiovascular agents[Title/Abstract]) OR (anticoagulant[Title/Abstract]) OR (anticoagulants[Title/Abstract]) OR (antiplatelet agent[Title/Abstract]) OR (antiplatelet agents[Title/Abstract]) OR (thrombolytic agent[Title/Abstract]) OR (thrombolytic agents[Title/Abstract]) OR (beta blocker[Title/Abstract]) OR (beta blockers[Title/Abstract]) OR (vasodilator[Title/Abstract]) OR (vasodilators[Title/Abstract])) AND ((artificial intelligence[Title/Abstract]) OR (machine learning[Title/Abstract]) OR (deep learning[Title/Abstract]) OR (support vector[Title/Abstract]) OR (random forest[Title/Abstract]) OR (neural network[Title/Abstract])))
AI / ML	(machine learning[Title/Abstract]) OR (artificial intelligence[Title/Abstract]) OR (deep learning[Title/Abstract]) OR (support vector[Title/Abstract]) OR (random forest[Title/Abstract]) OR (neural network[Title/Abstract])
