# Supplementary material for: Accelerating antibiotic discovery through artificial intelligence
Source: Commun Biol. 2021 Sep 9;4:1050. doi: 10.1038/s42003-021-02586-0 (PMC8429579; doi:10.1038/s42003-021-02586-0)
Supplement: Supplementary file 2 — Description of Additional Supplementary Files [file 42003_2021_2586_MOESM2_ESM.pdf]

Description of Additional Supplementary Files

File name: Supplementary Data 1

Description: Compacted file with 5 CSV files containing the raw PubMed data outputs visualized in Figure 3.
